# A novel triplex real-time PCR assay for the differentiation of lumpy skin disease virus, goatpox virus, and sheeppox virus

**DOI:** 10.3389/fvets.2023.1175391

**Published:** 2023-06-28

**Authors:** Wenlong Nan, Mingxia Gong, You Lu, Jinming Li, Lin Li, Hailong Qu, Chunju Liu, Ying Wang, Faxing Wu, Xiaodong Wu, Zhiliang Wang, Yiping Chen, Daxin Peng

**Affiliations:** ^1^China Animal Health and Epidemiology Center, Qingdao, China; ^2^College of Veterinary Medicine, Yangzhou University, Yangzhou, Jiangsu, China

**Keywords:** *Capripoxvirus*, detection, qPCR, differentiation, LSDV, SPPV, GTPV

## Abstract

**Introduction:**

Three members of *Capripoxvirus* (CaPV) genus, including lumpy skin disease virus (LSDV), goatpox virus (GTPV), and sheeppox virus (SPPV), are mentioned as notifiable forms by World Organization for Animal Health. These viruses have negatively impacted ruminant farming industry worldwide, causing great economic losses. Although SPPV and GTPV cause more severe clinical disease in only one animal species, they can transfer between sheep and goats. Both homologous and heterologous immunization strategies are used to protect animals against CaPVs. However, development of accurate and rapid methods to distinguish these three viruses is helpful for the early detection, disease surveillance, and control of CaPV infection. Therefore, we developed a novel triplex real-time PCR (qPCR) for the differentiation of LSDV, GTPV, and SPPV.

**Methods:**

Universal primers were designed to detect pan-CaPV sequences. Species-specific minor groove binder (MGB)-based probes were designed, which were labeled with FAM for LSDV, HEX for GTPV, and ROX for SPPV. The sensitivity, specificity, reproducibility, and ability of detecting mixed infections were evaluated for the triplex qPCR. Further, 226 clinical samples of the infection and negative controls were subjected to the triplex qPCR, and the results were verified using PCR-restriction fragment length polymorphism (PCR-RFLP) and sequencing methods for PRO30 gene.

**Results:**

The triplex qPCR could successfully distinguish LSDV, GTPV, and SPPV in one reaction, and the assay sensitivity was 5.41, 27.70, and 17.28 copies/μL, respectively. No cross-reactivity was observed with other viruses causing common ruminant diseases, including des petits ruminants virus, foot-and-mouth disease virus, bluetongue virus, ovine contagious pustular dermatitis virus, infectious bovine rhinotracheitis virus, and bovine viral diarrhea-mucosal disease virus. Inter-and intra-assay variabilities were < 2.5%. The results indicated that the triplex qPCR was highly specific, sensitive, and reproducible. Simulation experiments revealed that this assay could successfully distinguish two or three viruses in case of mixed infections without any cross-reaction. For clinical samples, the results were completely consistent with the results of PCR-RFLP and sequencing. This demonstrated that the assay was reliable for clinical application.

**Discussion:**

The triplex qPCR is a robust, rapid, and simple tool for identifying various types of CaPV as it can successfully distinguish LSDV, GTPV, and SPPV in one reaction. Furthermore, the assay can facilitate more accurate disease diagnosis and surveillance for better control of CaPV infection.

## 1. Introduction

Lumpy skin disease virus (LSDV), goatpox virus (GTPV), and sheeppox virus (SPPV) are members of the genus *Capripoxvirus* (CaPV). Symptoms of CaPV infection mainly include fever, enlarged lymph nodes, and nodules (or papules) on the skin and mucous membrane (1, 2). Sometimes, CaPV infection can lead to death; particularly GTPV and SPPV infections may cause 100% mortality in fully susceptible breeds of sheep and goats (2). Moreover, it can lead to economic losses due to the performance degradation in production, such as decreased milk yield, weight loss, skin damage, abortion in female animals, and temporary or permanent infertility in male animals. Due to the economic importance of cattle, sheep, and goat farming industries and potential of rapid transboundary spread of CaPVs, all CaPVs are in notifiable form as per World Organization for Animal Health (WOAH).

Previously, lumpy skin disease (LSD) mainly prevailed in Africa, Middle East, and south-eastern Europe (3, 4). From 2019 to 2021, LSD has spread rapidly in continental Asia, invading China, India, Vietnam, Thailand, Malaysia, and Mongolia. Prevention and control of LSD have been under focus in China and other Asian countries. Goatpox (GTP) and sheeppox (SPP) are widespread throughout Northern and Central Africa, Middle East, Central Asia, East Asia, and Southeast Asia (5). In China, GTP and SPP have been persistent epidemics in some provinces for years (6, 7).

Outbreaks of LSDV, GTPV, and SPPV have been occurring worldwide and have caused significant economic losses. LSDV mainly infects cattle and do not infect and transmit between sheep and goats (1). Strains of SPPV and GTPV can pass between sheep and goats, although most strains cause more severe clinical disease in only one animal species (2, 4). Control and eradication of CaPVs depend on vector control, early detection of outbreaks, restrictions on animal movement, and vaccination (8). Live attenuated vaccines are commercially available and mostly used to control CaPV infections. As the genomes of these three viruses are highly homologous, both homologous and heterologous immunization strategies are used in various countries (3). However, the protection conferred by heterologous vaccines for LSDV is not as effective as that conferred by homologous vaccines in animals (9–12). Thus, early detection and clear differentiation of these three viruses is important for selecting and implementing targeted prevention and control strategies. In the current study, we developed a novel triplex real-time quantitative PCR (qPCR) assay for sensitive, specific, robust, and fast differentiation of LSDV, GTPV, and SPPV in one reaction.

## 2. Materials and methods

### 2.1. Viruses and clinical samples

Lumpy skin disease virus/China/XJ/2019-1 and peste des petits ruminants virus (PPRV/China/2013) were isolated and identified at China Animal Health and Epidemiology Center (CAHEC). GTPV vaccine (AV41 strain), foot-and-mouth disease virus bivalent vaccine (FMDV Re-O/MYA98/JSCZ 2013 and Re-A/WH/09 strains), infectious bovine rhinotracheitis virus and bovine viral diarrhea-mucosal virus mixed vaccine (BVDV/NMG and IBRV/LY strains), and ovine contagious pustular dermatitis virus vaccine (ORFV/GO-BT15-30 strain) were purchased from the market. Nucleic acid (NA) of SPPV (GL strain) was provided by Lanzhou Veterinary Research Institute. NA of bluetongue virus (BTV) was provided by Institute of Military Veterinary Medicine. In total, 184 clinical samples from cattle and 42 clinical samples from goats or sheep suspected to be infected with CaPVs were provided by CAHEC and Animal Disease Prevention and Control Center of Inner Mongolia. The types of samples included whole blood, serum, saliva, and skin tissue. All the samples were stored at −70°C until further analysis.

### 2.2. Primer and probe designing

To design the candidate primers and probes, the complete genomic sequences of LSDV, GTPV, and SPPV obtained from GenBank were aligned using MegAlign software of Lasergene. A conserved region of the CaPV ORF091 gene (accession number: AF369026.1) was obtained via the designed similarity comparative algorithm approach. The primers and probes were designed using Primer Premier 5 and Beacon Designer 7.7. Universal primers were designed for all CaPV strains, and three minor groove binder (MGB)-based probes were designed to individually detect LSDV, GTPV, and SPPV. The probes were labeled with fluorescent dyes [6-carboxy fluorescein (FAM), hexachloro fluorescein (HEX), and carboxy-x-rhodamine (ROX) for detecting LSDV, GTPV and SPPV, respectively] on the 5′ end and with a compatible MGB quencher at the 3′ end. The sequences of primers and probes are given in Table 1. The primers and probes were synthesized by Ruibiotech (Qingdao, China).

**Table 1:** Primers and probes used in the triplex qPCR.

	Sequence(5′ → 3′)	Position on ORF091 (Accession numbers: AF369026.1)
091 F	CAACCAACAATAACTATTATCTGAA	35–59
091 R	TTTCTTCTCCTAAATCAGTTGC	143–161
091 LP	FAM-CGCTACGGGTACGT-MGB	77–91
091 GP	HEX-CGCCACGGATACGT-MGB	77–91
091 SP	ROX-TCCGCCACAGATATGT-MGB	75–91

### 2.3. NA extraction

The NA from whole blood, serum, saliva, virus, and other liquid samples was directly extracted with commercialized magnetic-bead-based viral DNA/RNA Extraction Kit (Tianlong, Xian, China) using automatic NA extractor (Tianlong, Xian, China). The skin tissue samples were initially homogenized and centrifuged, and the NA was extracted from the supernatant as per the method described above. The NA samples were stored at −70°C until further analysis.

### 2.4. Construction of positive controls

Positive control plasmids containing the target fragment of the ORF091 gene were constructed as follows. Overall, 2 μL NA of LSDV, GTPV, and SPPV was added to 25 μL reaction mixture containing 2× Platinum Super Green PCR MIX (Thermo Fisher Scientific, United States) and 500 nM of each 091F/091R primer. ddH_2_O was added to make up the volume to 50 μL. The conventional PCR conditions were 95°C for 5 min; 35 cycles of denaturation at 95°C for 30 s, annealing at 55°C for 30 s, extension at 72°C for 30 s; and final extension at 72°C for 5 min. The PCR products were recovered and ligated to pMD19-T vector. The concentration of the positive plasmids was estimated using NanoDrop one (Thermo Fisher Scientific, United States), and the copy number was determined as per the equation given below.

$$\begin{array}{c} \mathrm{copies of plasmid}=\left(\mathrm{concentration}\times 10^{-9}\times 6.02\times 10^{23}\right)\\ /\left(660\;\mathrm{Daltons}\;\mathrm{per}\;\mathrm{base}\times \mathrm{quantity of bases}\right) \end{array}$$

### 2.5. Triplex qPCR

In total, 2 μL of the sample NA was added to 10 μL reaction mixture containing 2× AceQ^®^ Universal U+ Probe Master Mix V2 (Vazyme, Nanjing, China) and 250 nM of each primer and each probe. ddH_2_O was added to make up the volume to 20 μL. The triplex qPCR was performed on the CFX 96™ real-time PCR machine (Bio-Rad, Hercules, CA, United States) with the following conditions: 37°C for 2 min, 95°C for 5 min, and 40 cycles of denaturation at 95°C for 10 s and annealing at 58°C for 30 s. The data acquisition was performed during the annealing step on three different channels including red for FAM, green for HEX, and orange for ROX.

### 2.6. Assay sensitivity

To determine the assay sensitivity of the triplex qPCR, 10-fold serially diluted positive control plasmids containing the ORF091 gene of LSDV, GTPV, and SPPV were tested to determine the lowest detection limit (LOD). The standard curves were generated. The efficiency of the assay was determined using the following calculation:

$$\mathrm{Efficiency}=10\;\left(-1/\mathrm{slope}\right)-1$$

### 2.7. Assay reproducibility

Assay reproducibility was determined by calculating the intra-and inter-assay coefficients of variation (CVs), using at least three replicates of each of the 10-fold serial dilutions of positive controls containing the ORF091 gene of LSDV, GTPV, and SPPV.

### 2.8. Assay specificity

To determine the specificity of the triplex qPCR, the assay was initially performed using virus-derived NA templates of LSDV, GTPV, and SPPV. Further, the assay was performed using NA templates of non-CaPV pathogens, including FMDV, PPRV, IBRV, BTV, BVDV, and ORFV. To evaluate the assay specificity in case of clinically mixed infections, the assay was performed using virus-derived NA templates of various viruses mixed in 1:1 ratio.

### 2.9. Evaluation of simulated mixed viral samples

Three positive control plasmids (of concentration 10^5^ copies/μL) were mixed in various proportions and were used to simulate clinical samples of double and triple infection. The ability of the triplex qPCR to differentiate mixed infection samples was assessed. Three replicates of each proportion were used, and the CVs were calculated.

### 2.10. Evaluation of clinical samples using triplex qPCR

The detection power and usability of the assay was evaluated using NA extracted from blood, serum, saliva, and skin tissue collected from cattle, goats, and sheep with suspected CaPVs infection. All samples were validated using PCR-restriction fragment length polymorphism (PCR-RFLP) for PRO30 gene as described previously (13). Furthermore, all positive PCR products were sequenced and aligned using NCBI server.

## 3. Results

### 3.1. Primer and probe designing

**Figure 1:**
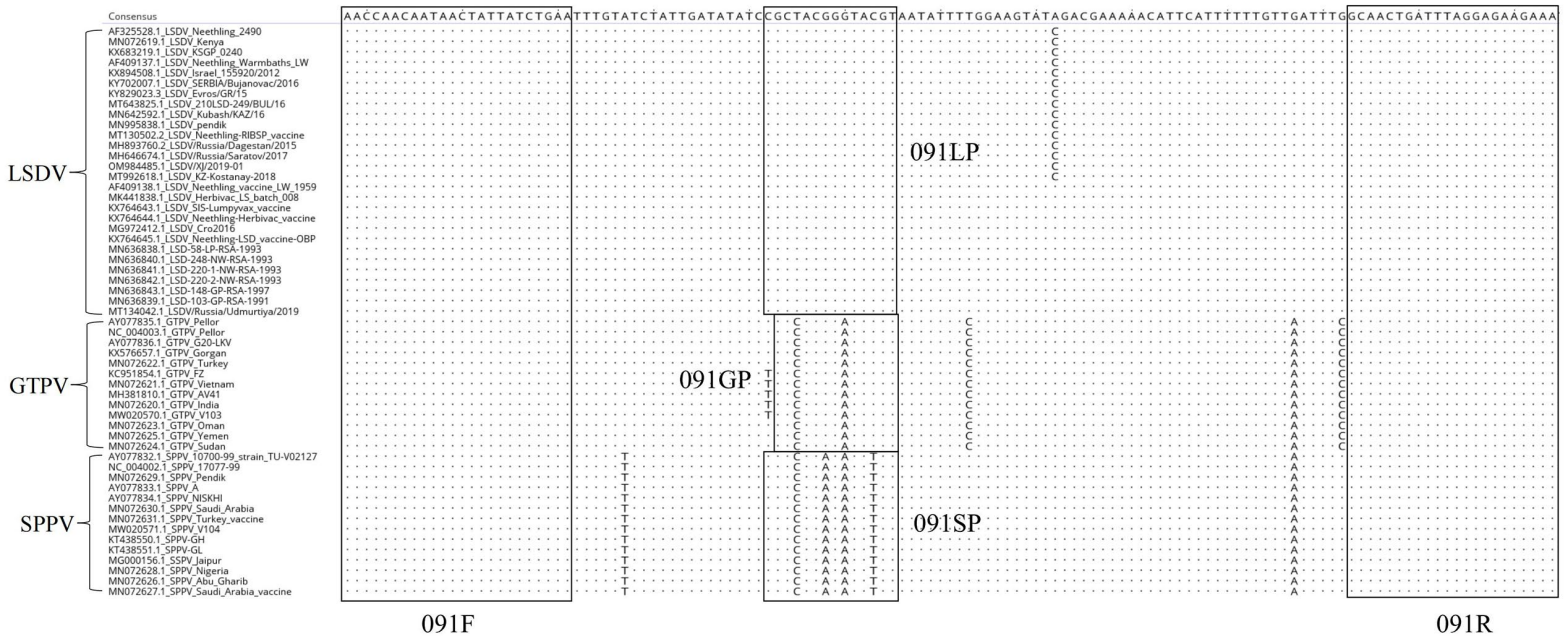
Alignment of ORF091 sequences of lumpy skin disease virus (LSDV), goatpox virus (GTPV), and sheeppox virus (SPPV).

The whole genomic sequences of various strains of GTPV, SPPV, and LSDV were downloaded from GenBank and compared using the MegAlign software. The primers designed in this study were conserved and suitable for the amplification of various strains of CaPV based on BLAST hits, whereas the probes were unique for LSDV, GTPV, and SPPV (Figure 1).

### 3.2. Construction of positive controls

Three plasmids containing related ORF091 gene fragments were successfully constructed and used as the positive controls for LSDV, GTPV, and SPPV, respectively. The concentration of LSDV, GTPV, and SPPV positive controls was 11.82 (5.41 × 10^9^ copies/μL), 60.48 (2.77 × 10^10^ copies/μL), and 37.52 (1.73 × 10^10^ copies/μL) ng/μL, respectively. These positive controls were further used for evaluation of the assay.

### 3.3. Linearity and LOD of the assay

**Figure 2:**
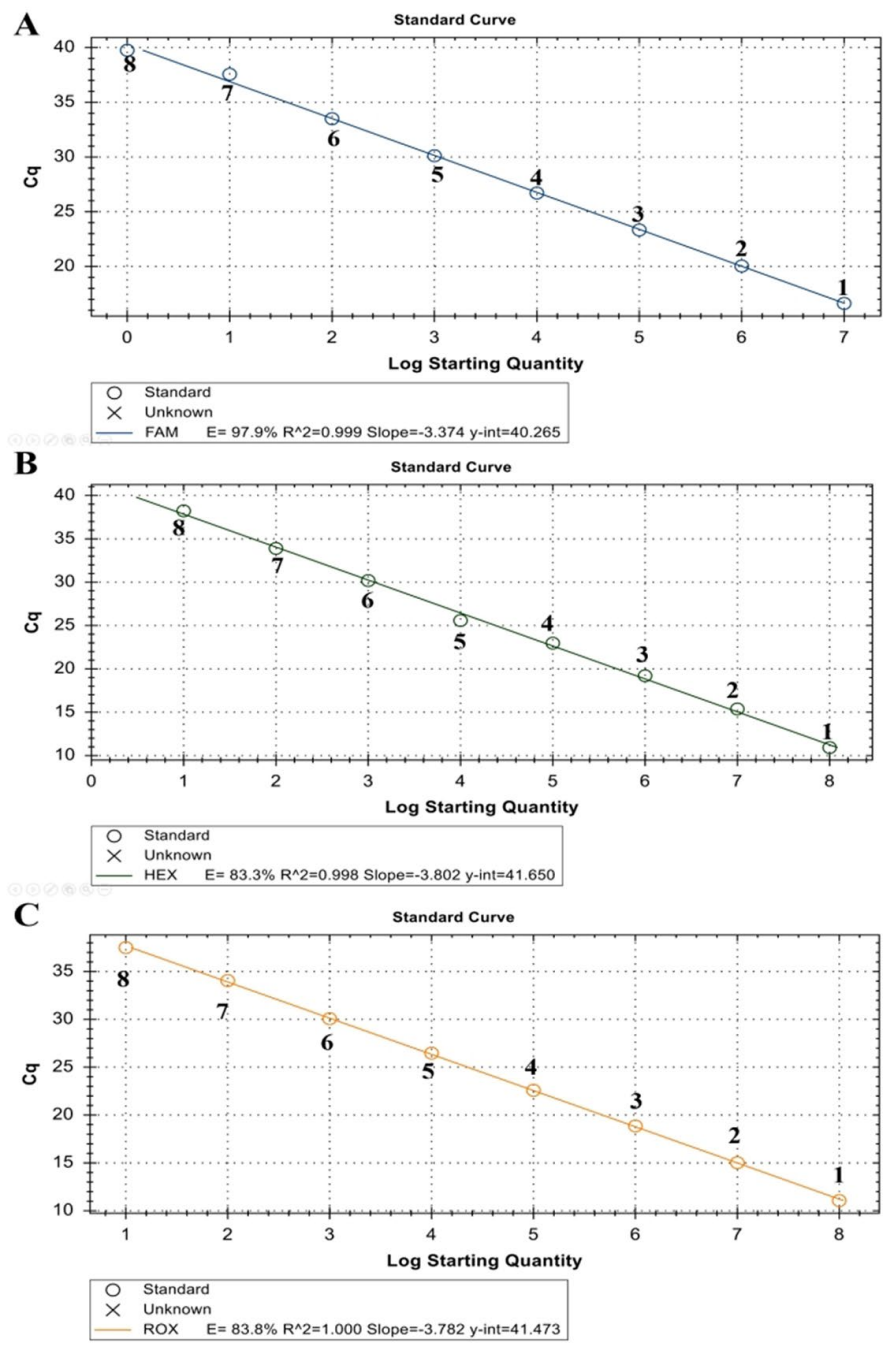
Standard curve of ORF091 positive controls in the triplex qPCR. **(A)** LSDV ORF091 positive control; **(B)** GTPV ORF091 positive control; **(C)** GTPV ORF091 positive control. Dilution factor 1: 10^2^; 2: 10^3^; 3: 10^4^; 4: 10^5^; 5: 10^6^; 6: 10^7^; 7: 10^8^; and 8: 10^9^.

**Figure 3:**
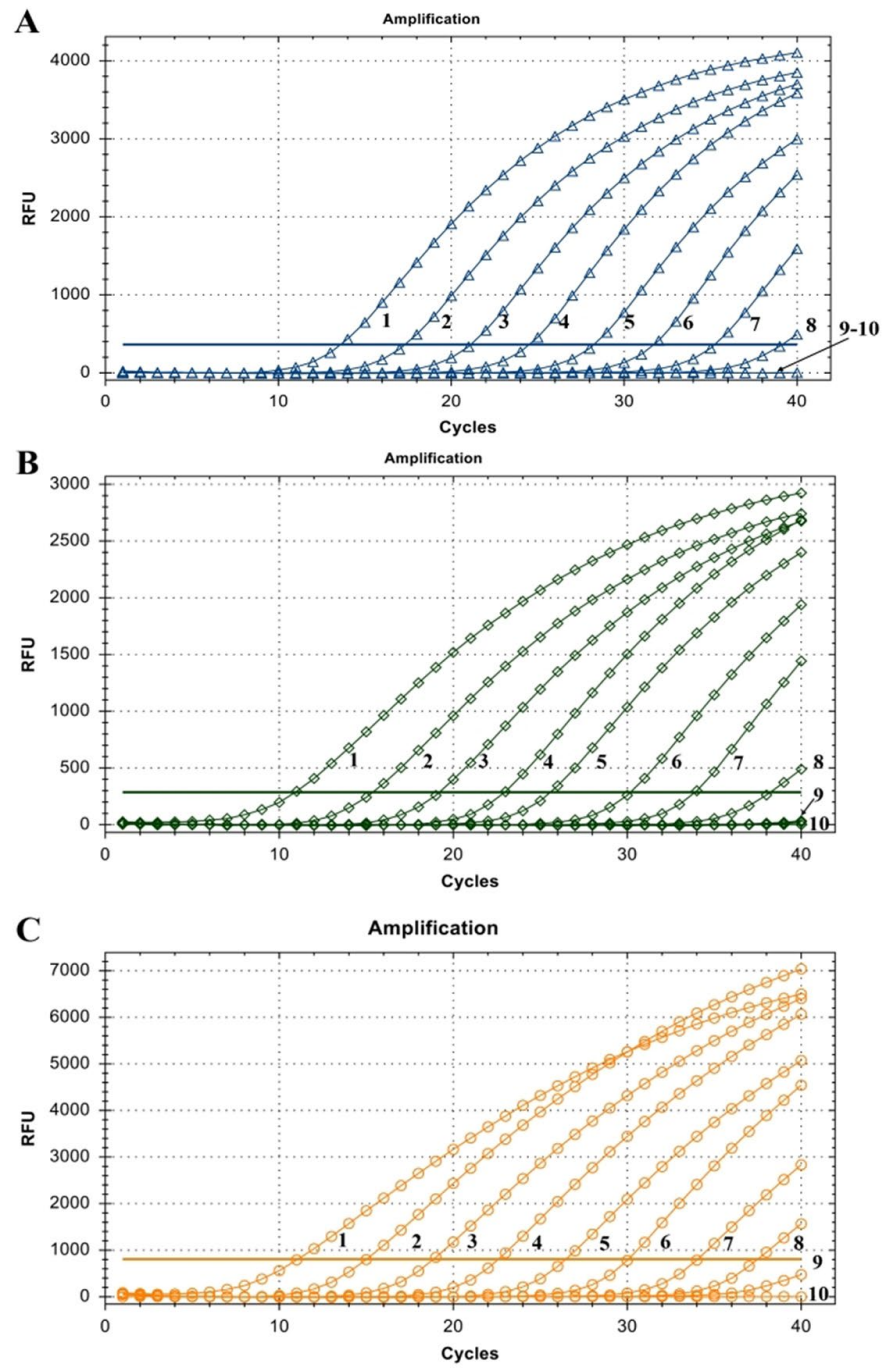
Assay sensitivity of the triplex qPCR. **(A)** LSDV ORF091 positive control; **(B)** GTPV ORF091 positive control; **(C)** GTPV ORF091 positive control. Dilution factor 1: 10^2^; 2: 10^3^; 3: 10^4^; 4: 10^5^; 5: 10^6^; 6: 10^7;^ 7: 10^8^; 8: 10^9^; 9: 10^10^; 10: ddH_2_O. FAM, HEX, and ROX fluorescence signals were represented by △, ◇, and ○, respectively.

The linearity of the assay was established by amplifying 10-fold serial dilutions of positive controls. The resulting Cq value was plotted against the log input copy number. Using 10^2^ to 10^9^ dilutions, the efficiencies were determined as 97.9, 83.3, and 83.8% for LSDV, GTPV, and SPPV, respectively (Figure 2). The correlation coefficient (*R*^2^) values were > 0.998 (Figure 2). The LOD was 5.41, 27.70, and 17.28 copies/μL for LSDV, GTPV, and SPPV, respectively (Figure 3). The results revealed that the assay was sensitive and could accurately estimate sample concentration in a large-scale linear range.

### 3.4. Cross-reactivity between homologous viruses or viruses causing other common diseases

**Figure 4:**
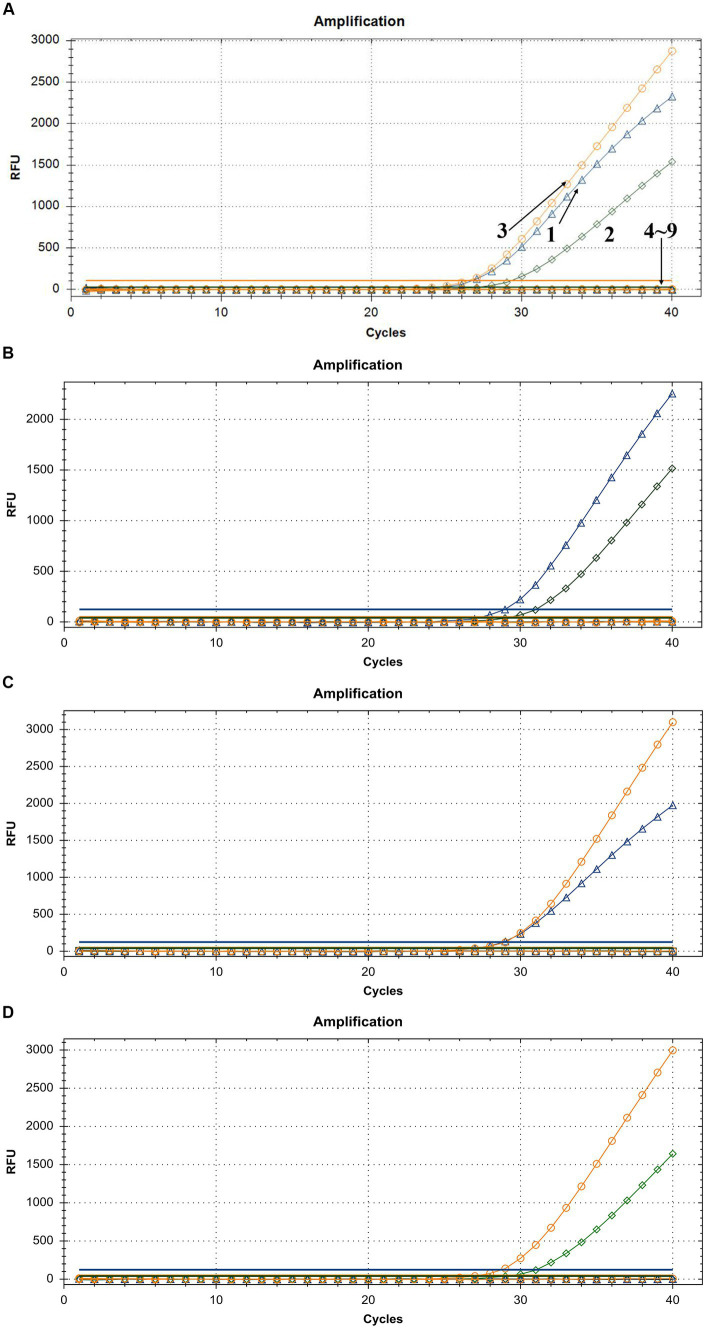
Assay specificity of the triplex qPCR. **(A)** 1: LSDV; 2: GTPV; 3: SPPV; 4: BTV; 5: FMDV; 6: BVDV and IBRV; 7: ORFV; 8: PPRV; and 9: ddH_2_O. **(B)** LSDV + GTPV. **(C)** LSDV + SPPV. **(D)** GTPV+SPPV. FAM, HEX, and ROX fluorescence signals were represented by △, ◇, and ○, respectively.

To demonstrate the assay specificity in homologous viruses, NAs extracted from LSDV, GTPV, and SPPV were evaluated, and the concentrations were 24.35, 11.82, and 20.32 ng/μL, respectively. The LSDV sample produced a strong FAM fluorescence with no HEX or ROX fluorescence. The GTPV sample produced a strong HEX fluorescence with no FAM or ROX fluorescence, and the SPPV sample produced a strong ROX fluorescence with no FAM or HEX fluorescence (Figure 4A). To distinguish LSDV, GTPV, and SPPV from other viruses causing common ruminant diseases, the assay was tested using FMDV, PPRV, IBRV, BTV, BVDV, and ORFV. The samples of these other viruses exhibited negative FAM, HEX, or ROX fluorescence (Figure 4A). In case of mixed-virus-derived NA, the LSDV+GTPV sample produced strong FAM and HEX fluorescence with no ROX fluorescence (Figure 4B); the LSDV+SPPV sample produced strong FAM and ROX fluorescence with no HEX fluorescence (Figure 4C), and the GTPV+SPPV sample produced strong HEX and ROX fluorescence with no FAM fluorescence (Figure 4D). Neither false positivity nor interference was observed among the three fluorophore signals, indicating high specificity.

### 3.5. Inter-and intra-assay variability

The assay reproducibility was evaluated using 10-fold serial dilutions of positive controls. The CVs are summarized in Table 2. The CVs between runs were mostly in the range of 0.18–2.31% for LSDV, 0.16–1.78% for GTPV, and 0.37–2.29% for SPPV (Table 2). The assay exhibited a high reproducibility in inter-and intra-assay testing.

**Table 2:** Inter-and Intra-assay variability of the triplex qPCR.

Copies/μL of LSDV plasmid	Inter-CV%	Intra-CV%	Copies/μL of GTPV plasmid	Inter-CV%	Intra-CV%	Copies/μL of SPPV plasmid	Inter-CV%	Intra-CV%
5.41 × 10^6^	2.31%	1.03%	2.77 × 10^7^	1.78%	1.22%	1.73 × 10^7^	0.40%	0.91%
5.41 × 10^5^	0.88%	1.73%	2.77 × 10^6^	0.16%	1.26%	1.73 × 10^6^	1.95%	1.60%
5.41 × 10^4^	1.28%	1.36%	2.77 × 10^5^	0.27%	0.97%	1.73 × 10^5^	0.99%	0.86%
5.41 × 10^3^	0.78%	1.33%	2.77 × 10^4^	1.33%	1.22%	1.73 × 10^4^	2.29%	0.63%
5.41 × 10^2^	0.97%	0.84%	2.77 × 10^3^	0.37%	0.67%	1.73 × 10^3^	0.77%	1.12%
5.41 × 10^1^	2.30%	0.18%	2.77 × 10^2^	1.78%	1.22%	1.73 × 10^2^	0.37%	0.47%

### 3.6. Simulation of mixed infections

The artificially mixed plasmids that simulated clinical double and triple mixed infections were analyzed using the triplex qPCR. The assay successfully distinguished two or three types of plasmids in various mixed schemes without any cross-reaction (Table 3). The CVs of the assay for detecting simulated double and triple mixed infection were < 2.68 and < 3.92%, respectively (Table 3).

**Table 3:** Mean Ct values and CV% of the triple qPCR for testing artificial mixtures of control plasmids.

Mixed volumes of LSDV,GTPV/SPPV-positive plasmids	Mean Ct for testing LSDV	Mean Ct for testing GTPV	Mean Ct for testing SPPV	Intra-CV%
1/9/−	32.25 ± 0.09	29.44 ± 0.21	none	0.42%/1.01%/0%
3/7/−	28.82 ± 0.29	29.81 ± 0.09	none	1.40%/0.43%/0%
5/5/−	28.28 ± 0.54	28.89 ± 0.94	none	2.68%/1.88%/0%
7/3/−	27.68 ± 0.13	30.90 ± 0.14	none	0.69%/0.60%/0%
9/1/−	26.68 ± 0.22	32.40 ± 0.37	none	1.19%/1.59%/0%
−/1/9	none	34.43 ± 0.39	27.48 ± 0.29	0%/1.58%/1.47%
−/3/7	none	31.67 ± 0.25	27.82 ± 0.26	0%/1.12%/1.32%
−/5/5	none	30.53 ± 0.24	28.17 ± 0.20	0%/1.09%/1.00%
−/7/3	none	30.37 ± 0.15	28.90 ± 0.23	0%/0.72%/1.13%
−/9/1	none	29.44 ± 0.20	31.01 ± 0.13	0%/0.96%/0.57%
1/−/9	31.20 ± 0.36	none	27.36 ± 0.15	1.63%/0%/0.75%
3/−/7	29.16 ± 0.03	none	28.66 ± 0.02	0.12%/0%/0.10%
5/−/5	28.05 ± 0.01	none	28.91 ± 0.05	0.05%/0%/0.22%
7/−/3	27.57 ± 0.21	none	29.27 ± 0.04	1.05%/0%/0.19%
9/−/1	27.25 ± 0.06	none	31.87 ± 0.52	0.34%/0%/2.29%
5/1/9	28.78 ± 0.10	36.85 ± 0.05	30.72 ± 0.79	0.49%/0.19%/3.66%
5/3/7	27.95 ± 0.27	32.72 ± 0.07	29.25 ± 0.81	1.34%/0.30%/3.92%
5/5/5	28.30 ± 0.08	31.45 ± 0.34	29.48 ± 0.64	0.37%/1.51%/3.07%
5/7/3	28.66 ± 0.18	30.36 ± 0.03	29.47 ± 0.16	0.86%/0.14%/0.74%
5/9/1	28.57 ± 0.02	29.92 ± 0.18	30.05 ± 0.18	0.12%/0.87%/0.85%
1/5/9	29.39 ± 0.16	30.95 ± 0.16	31.24 ± 0.13	0.77%/0.71%/0.57%
3/5/7	28.77 ± 0.14	31.15 ± 0.04	28.72 ± 0.33	0.66%/0.16%/1.62%
5/5/5	28.55 ± 0.29	31.22 ± 0.05	28.56 ± 0.04	1.44%/0.20%/0.17%
7/5/3	28.22 ± 0.15	32.05 ± 0.11	28.05 ± 0.05	0.73%/0.49%/0.28%
9/5/1	28.11 ± 0.21	33.17 ± 0.06	27.65 ± 0.42	1.06%/0.26%/2.15%
1/9/5	28.94 ± 0.07	33.52 ± 0.12	27.09 ± 0.32	0.34%/0.51%/1.67%
3/7/5	29.07 ± 0.17	30.86 ± 0.37	27.74 ± 0.44	0.83%/1.67%/2.24%
5/5/5	28.36 ± 0.42	31.60 ± 0.42	28.07 ± 0.67	2.09%/1.88%/3.38%
7/3/5	28.18 ± 0.11	31.09 ± 0.05	29.52 ± 0.38	0.53%/0.23%/1.82%
9/1/5	27.44 ± 0.47	31.37 ± 0.17	29.32 ± 0.33	2.45%/0.77%/1.59%
5/−/−	25.62 ± 0.15	none	none	0.83%/0%/0%
−/5/−	none	27.40 ± 0.18	none	0%/0.96%/0%
−/−/5	none	none	24.15 ± 0.16	0%/0%/0.94%
−/−/−	none	none	none	0%/0%/0%

### 3.7. Virus detection in clinical samples

The detection of viruses in 226 clinical samples (including 95 samples infected with LSDV, six samples infected with GTPV, seven samples infected with SPPV, and 118 negative samples) exhibited a detection rate of 100%. This was consistent with the results obtained by PCR-PFLP and sequencing methods.

## 4. Discussion

*Capripoxvirus* infection in ruminants causes significant morbidity and mortality in Africa, Middle East, and Asia; therefore, it has a great economic importance (14). Currently, all CaPVs are ranked as class II animal diseases in China. Attenuated GTPV strain AV41 is widely used to immunize cattle, goats, and sheep to prevent CaPV. Combined with movement control, quarantine supervision, sterilization, insect control, and other measures, the number of CaPV outbreaks has greatly reduced, particularly LSDV outbreaks in China in recent years.

Lumpy skin disease virus infection seems to be restricted to cattle and large wild ruminants. SPPV and GTPV infect mainly small domestic and wild ruminants, which may share similar geographical locations and same animal species. Both lead to the formation of generalized or localized cutaneous lesions (14). For controlling CaPV infection, immunization is a critical measure and live attenuated vaccines are being used for years. Globally, sheep, goats, and cattle are mostly vaccinated with Romania, Bakirkoy, Yugoslavian RM65, KSGP O-240, and KSGP O-180 strains; Gorgan, Mysore, Uttarkashi, and KSGP O-240 strains; and Neethling, KSGP O-240 and KSGP O-180, Romania, Bakirkoy, and Gorgan strains (3), respectively, which are homologous and heterologous immunization strategies. Thus, development of accurate and rapid methods to distinguish these three viruses is helpful for the early detection, surveillance, and control of CaPV infection, as well as for the differentiation of wild-type strains and vaccine strains when heterologous immunization strategies are used.

*Capripoxviruses* are closely related, with genomic sequence identities ranging from 96% (between species) to 99% (between the strains of same species) (15). It is difficult to serologically distinguish these three viruses. For the viral detection, conventional PCR, qPCR, and HRM methods have been developed and validated (13, 14, 16–19). Conventional PCR involves gel electrophoresis. HRM assay strongly depends on PCR conditions, instruments, and dyes, and the accuracy is critically dependent on the resolution of the instrument (20, 21). In recent years, because of the outbreaks of African swine fever and Coronavirus disease 2019, qPCR application is greatly promoted and widely accepted, particularly in China (22–25). qPCR is considered one of the most useful tools for clinical molecular detection, which is reliable, sensitive, and cost-effective.

In the present study, we developed a new MBG-based triplex qPCR that can successfully distinguish LSDV, GTPV, and SPPV in one reaction. The LOD was 5.41, 27.70, and 17.28 copies/μL for LSDV, GTPV, and SPPV, respectively. The probes were BLAST searched and found to be specific for the related species only. The assay exhibited no cross-reactivity with the viruses causing common ruminant-related diseases including PPRV, FMDV, ORFV, BTV, IBRV, and BVDV. Furthermore, the CVs of inter-and intra-assay were <2.5%. Through simulation experiments, the assay successfully distinguished CaPVs in case of mixed infections without any cross-reaction. The results indicated the triplex qPCR assay is highly specific, sensitive, and reproducible for distinguishing LSDV, GTPV, and SPPV. The assay was evaluated using clinical samples, and the results were completely consistent with the results of PCR-PFLP and sequencing methods. This indicated that the assay is reliable for clinical application.

Therefore, the assay provided a robust, rapid, and simple tool for the differentiation of LSDV, GTPV, and SPPV and facilitated more accurate disease diagnosis and surveillance for better control of CaPV.

## Data availability statement

The original contributions presented in the study are included in the article/supplementary material, further inquiries can be directed to the corresponding authors.

## Author contributions

YC, DP, and WN were responsible for the experimental design and drafting the manuscript. MG, YL, JL, LL, HQ, CL, YW, FW, XW, and ZW performed the experiments and analyzed data. All authors contributed to the article and approved the submitted version.

## Funding

This work was supported by National Key Research and Development Program (No. 2022YFD1800500).

## Conflict of interest

The authors declare that the research was conducted in the absence of any commercial or financial relationships that could be construed as a potential conflict of interest.

## Publisher’s note

All claims expressed in this article are solely those of the authors and do not necessarily represent those of their affiliated organizations, or those of the publisher, the editors and the reviewers. Any product that may be evaluated in this article, or claim that may be made by its manufacturer, is not guaranteed or endorsed by the publisher.

## References

[1] World Organization of Animal Health (2019) Manual of diagnostic tests and vaccines, Ch. 3.04.12. Available at: https://www.oie.int/fileadmin/Home/eng/Health_standards/tahm/3.04.12_LSD.pdf

[2] World Organization of Animal Health (2019) Manual of diagnostic tests and vaccines. Chapter 3.07.12 Available at: https://www.oie.int/fileadmin/Home/eng/Health_standards/tahm/3.07.12_S_POX_G_POX.pdf

[3] Hamdi J Munyanduki H Omari Tadlaoui K El Harrak M Fassi FO. Capripoxvirus infections in ruminants: a review. Microorganisms. (2021) 9:902. doi: 10.3390/microorganisms9050902, PMID: 33922409 PMC8145859

[4] Tuppurainen E Venter EH Shisler JL Gari G Mekonnen GA Juleff N. Review: Capripoxvirus diseases: current status and opportunities for control. Transbound Emerg Dis. (2017) 64:729–45. doi: 10.1111/tbed.12444, PMID: 26564428 PMC5434826

[5] Hamdi J Bamouh Z Jazouli M Boumart Z Tadlaoui KO Fihri OF. Experimental evaluation of the cross-protection between Sheeppox and bovine lumpy skin vaccines. Sci Rep. (2020) 10:8888. doi: 10.1038/s41598-020-65856-7, PMID: 32483247 PMC7264126

[6] Lu G Xie J Luo J Shao R Jia K Li S. Lumpy skin disease outbreaks in China, since 3 august 2019. Transbound Emerg Dis. (2021) 68:216–9. doi: 10.1111/tbed.13898, PMID: 33119963

[7] Li L Qi C Li J Nan W Wang Y Chang X. Quantitative real-time PCR detection and analysis of a lumpy skin disease outbreak in Inner Mongolia Autonomous Region. Front Vet Sci. (2022) 9:936581. doi: 10.3389/fvets.2022.936581, PMID: 35958309 PMC9362877

[8] Tuppurainen ES Galon N. Lumpy skin disease: Current situation in Europe and neighbouring regions and necessary control measures to halt the spread in south-East Europe. Oie Reg. Comm. (2016).

[9] Yeruham I Nir O Braverman Y Davidson M Grinstein H Haymovitch M. Spread of lumpy skin disease in Israeli dairy herds. Vet Rec. (1995) 137:91–3. doi: 10.1136/vr.137.4.91, PMID: 8533249

[10] Ayelet G Abate Y Sisay T Nigussie H Gelaye E Jemberie S. Lumpy skin disease: preliminary vaccine efficacy assessment and overview on outbreak impact in dairy cattle at Debre Zeit, Central Ethiopia. Antivir Res. (2013) 98:261–5. doi: 10.1016/j.antiviral.2013.02.008, PMID: 23428671

[11] Ben-Gera J Klement E Khinich E Stram Y Shpigel NY. Comparison of the efficacy of Neethling lumpy skin disease virus and x10RM65 sheep-pox live attenuated vaccines for the prevention of lumpy skin disease—the results of a randomized controlled field study. Vaccine. (2015) 33:4837–42. doi: 10.1016/j.vaccine.2015.07.071, PMID: 26238726

[12] Calistri P DeClercq K Gubbins S Klement E Stegeman A Cortiñas Abrahantes J. Lumpy skin disease: III. Data Coll Analy EFSA J. (2019) 17:e05638. doi: 10.2903/j.efsa.2019.5638, PMID: 32626261 PMC7009259

[13] Wang H Kong Y Mei L Lv J Wu S Lin X. Multiplex real-time PCR method for simultaneous detection and differentiation of Goatpox virus, Sheeppox virus, and lumpy skin disease virus. J AOAC Int. (2021) 104:1389–93. doi: 10.1093/jaoacint/qsab040, PMID: 33769495

[14] Gelaye E Mach L Kolodziejek J Grabherr R Loitsch A Achenbach JE. A novel HRM assay for the simultaneous detection and differentiation of eight poxviruses of medical and veterinary importance. Sci Rep. (2017) 7:42892. doi: 10.1038/srep42892, PMID: 28216667 PMC5316968

[15] Tulman ER Afonso CL Lu Z Zsak L Sur JH Sandybaev NT. The genomes of sheeppox and goatpox viruses. J Virol. (2002) 76:6054–61. doi: 10.1128/jvi.76.12.6054-6061.2002, PMID: 12021338 PMC136203

[16] Lamien CE le Goff C Silber R Wallace DB Gulyaz V Tuppurainen E. Use of the Capripoxvirus homologue of vaccinia virus 30kDa RNA polymerase subunit (RPO30) gene as a novel diagnostic and genotyping target: development of a classical PCR method to differentiate goat poxvirus from sheep poxvirus. Vet Microbiol. (2011) 149:30–9. doi: 10.1016/j.vetmic.2010.09.038, PMID: 21115310

[17] Agianniotaki EI Chaintoutis SC Haegeman A Tasioudi KE de Leeuw I Katsoulos P. Development and validation of a TaqMan probe-based real-time PCR method for the differentiation of wild type lumpy skin disease virus from vaccine virus strains. J Virol Methods. (2017) 249:48–57. doi: 10.1016/j.jviromet.2017.08.011, PMID: 28837841

[18] Wolff J Beer M Hoffmann B. Probe-based real-time qPCR assays for a reliable differentiation of Capripox virus species. Microorganisms. (2021) 9:765. doi: 10.3390/microorganisms9040765, PMID: 33917525 PMC8067474

[19] Chibssa TR Settypalli TBK Berguido FJ Grabherr R Loitsch A Tuppurainen E. An HRM assay to differentiate Sheeppox virus vaccine strains from Sheeppox virus field isolates and other Capripoxvirus species. Sci Rep. (2019) 9:6646. doi: 10.1038/s41598-019-43158-x, PMID: 31040355 PMC6491823

[20] Wittwer CT. High-resolution DNA melting analysis: advancements and limitations. Hum Mutat. (2009) 30:857–9. doi: 10.1002/humu.20951, PMID: 19479960

[21] Herrmann MG Durtschi JD Bromley LK Wittwer CT Voelkerding KV. Amplicon DNA melting analysis for mutation scanning and genotyping: cross-platform comparison of instruments and dyes. Clin Chem. (2006) 52:494–503. doi: 10.1373/clinchem.2005.063438, PMID: 16423901

[22] Gao Q Feng Y Yang Y Luo Y Gong T Wang H. Establishment of a dual real-time PCR assay for the identification of African swine fever virus genotypes I and II in China. Front Vet Sci. (2022) 9:882824. doi: 10.3389/fvets.2022.882824, PMID: 35720851 PMC9198542

[23] Yang H Peng Z Song W Zhang C Fan J Chen H. A triplex real-time PCR method to detect African swine fever virus gene-deleted and wild type strains. Front Vet Sci. (2022) 9:943099. doi: 10.3389/fvets.2022.943099, PMID: 36187818 PMC9521421

[24] Wang Y Xu L Noll L Stoy C Porter E Fu J. Development of a real-time PCR assay for detection of African swine fever virus with an endogenous internal control. Transbound Emerg Dis. (2020) 67:2446–54. doi: 10.1111/tbed.13582, PMID: 32306531

[25] Guo J Ge J Guo Y. Recent advances in methods for the diagnosis of Corona virus disease 2019. J Clin Lab Anal. (2022) 36:e24178. doi: 10.1002/jcla.24178, PMID: 34921443 PMC8761393

